# Dairy calves provided with environmental enrichment are more active, playful and have fewer feeding interruptions

**DOI:** 10.1038/s41598-025-88129-7

**Published:** 2025-02-04

**Authors:** Francesca Occhiuto, Jorge A. Vázquez-Diosdado, Matthew Thomas, Emma R. Gayner, Andrew J. King, Jasmeet Kaler

**Affiliations:** 1 School of Veterinary Medicine and Science, University of Nottingham, Sutton Bonington Campus, Leicestershire, LE12 5RD UK; 2 Department of Biosciences, Faculty of Science and Engineering, Swansea University, Singleton Park Campus, Swansea, SA2 8PP UK

**Keywords:** Animal behaviour, Behavioural methods

## Abstract

Concerns for farm animal welfare have led to the use of environmental enrichment to stimulate natural behaviours and promote positive emotions. In cattle, the provision of brushes is sometimes recommended but their use in calves and the effects they may have are not well established. The use of precision technologies enables the collection of detailed behavioural data that can be used as welfare indicators. Here we use ultra-wideband location sensors to measure activity and play, along with automatic milk feeders to measure feeding. We assessed the effects of stationary brushes on the behaviour of 226 dairy calves for up to 72 days. Half of the calves had access to the brushes for half of the experimental period. Using a mixed-effects linear model we showed that when brushes were present calves had significantly higher activity, fed slower, had fewer interruptions in their meals and spent less time around the feeder, suggesting reduced competition. Furthermore, calves that had access to brushes during the trial were more active and playful, even on days when the brushes were not available, compared to the control group. This finding indicates for the first time that enrichment may have a lasting effect on calf behaviour and welfare.

## Introduction

Promoting positive welfare in farm animals is a shared goal among scientists^1–3^, farmers^4,5^ and consumers^5,6^. In indoor housing systems, farm animals often experience relatively barren and more predictable environments compared to outdoor settings, leading to reduced opportunities for interactions with the physical environment and potential for the development of stereotypic behaviours^7^. Introducing environmental enrichment for animals housed indoors can stimulate natural behaviours and, in certain cases, directly evoke positive emotions in the animals^7–9^.

Dairy calves are a central part of the production of milk and dairy products consumed by millions of people worldwide^10^. Calves raised for dairy purposes are typically housed indoors, and their welfare is critical from an ethical perspective, and is linked with animal health, productivity, and farm profitability^11^. There is a growing recognition of the need to enhance the welfare of farm animals using environmental enrichment^7,8,12,13^. Brushes are often used as a form of environmental enrichment for indoor-housed dairy cows^14^ and steers^12^, providing a type of tactile enrichment allowing animals to mimic natural behaviours such as rubbing on trees to scratch or groom^15^. Cows are motivated to use brushes^9^ and their presence increases grooming time and reduces the number of idling events^8^. In steers, individuals show a decrease in stereotypic and aggressive behaviours, such as bar licking and headbutting, suggesting improved welfare when provided with brushes^12^. There are potential secondary benefits to providing brushes too, with increased milk yield reported for cows using brushes^16^. The effects of brushes on dairy calf behaviour are less frequently studied^17–19^, but the evidence so far indicates that calves provided with brushes use them for grooming and interact with them more than with other enrichment items^18^, and that the presence of brushes can result in decreased abnormal oral behaviours such as non-nutritive sucking in calves^17^, which may indicate reduced boredom.

Further insight into the potential effects of environmental enrichment may be achieved using automated data collection methods. Wearable sensors are increasingly common in dairy farms, as well as automated feeding systems for calves, which combined collect a large amount of data and can be used to monitor behaviour in detail^20–23^. Sensors have also been used to detect individual differences in behaviour between calves, demonstrating the existence of personality types in cattle^22,24,25^. Automated behavioural data collection therefore provides opportunity to precisely measure within and between individual differences in movement^22,26^, and feeding behaviours^24,25^, and explore behavioural responses to enrichment, which are yet to be studied in cattle (but see differences in brush use based on cow dominance^27^).

Here, we used automated data collection in the form of ultra-wideband location sensors^21,22,26^ and automatic calf feeders to investigate the effects of stationary brushes on the behaviour of dairy calves. We monitored the movement and feeding behaviours of 226 calves half of which were provided with three stationary brushes for half of the experimental time. We hypothesised that the presence of brushes would have positive effects on calf activity, play and feeding behaviours. Given that adult cattle and calves are attracted towards brushes^9^ and their presence reduces idling behaviour^8^ we first tested calf space use on days with and without the brushes present. We expected that calves would use the space where the brushes were located more on days when the brushes were present (prediction 1). Because the enrichment properties of objects can diminish with time^28^ and calves show personality differences in activity and movement^22,26^, we also tested for any decline in the use of space where brushes were located (prediction 2), and for any inter-individual differences in the time calves spend near the brushes (prediction 3).

Because we found larger differences in space use, but limited evidence of habituation or personality effects, we proceeded to test more specific behavioural changes. We expected higher levels of activity in the presence of brushes due to the calves walking to and from the brushes to use them, and we also expected reduced residence time as the presence of an additional element to interact with would lower the time spent in the same location (prediction 4). We also expected more play behaviour, which is often used as an indicator of welfare^29,30^ and was previously shown to be affected by a combination of enrichment items^18^. Regarding feeding, socially housed calves fed through automatic feeders experience feeding competition, which can lead to reduced milk consumption^31^. We expected that the attraction of the novel brush resource (above) would reduce interest in the feeder^32^, reducing time spent around the feeder on days when the brushes were present (prediction 5)^17^ which may reduce social interference around the feeder and lead to positive effects on feeding behaviour when brushes were present, such as reduced drinking speed and fewer unentitled visits (prediction 6). Because environmental enrichment not only affects behaviour when present but can result in long-term effects^33^ we also tested if and how any effects change over time by comparing the activity of our experimental group with calves that were reared in the same way but without the introduction of the brushes at any point. We expected any effects we identified (see above) would also be present on days when the brushes were absent compared to calves that never encountered them (prediction 7).

## Methods

### Ethical approval

The study was approved by the Ethics Committee of the School of Veterinary Medicine and Science, University of Nottingham (unique reference number 1481150603). All methods were performed in accordance with the relevant guidelines and regulations and are reported in accordance with the ARRIVE guidelines^34^.

### Animals, housing and farm management

The study took place at the Centre for Dairy Science Innovation at the University of Nottingham, UK, between July 2021 and April 2024. 226 Holstein Friesian dairy heifer calves were studied between June 2021 and January 2024, divided in 16 cohorts of up to 16 calves (mean = 14.1, SD = 1.8) as per normal farm management (details of the number of calves per cohort are in the supplementary material Table S1). The first 8 cohorts (125 calves) were raised without any enrichment while the following 8 cohorts (101 calves) received the experimental addition of brushes. All the calves were housed in pairs at birth and a cohort was formed by a minimum of 5 and a maximum of 8 pairs that were closest in age. When the youngest calf of the cohort was at least 15 days of age the cohort was moved to one of two adjacent straw-bedded trial pens (6 m × 10 m), where they stayed for 7–11 weeks. Each pen was fitted with an automated feeder which allocated up to 2 L every 2 h up to an initial entitlement of 10 L of milk replacer to each calf through RFID recognition as per farm usual farm management. After 38 days of full entitlement all the calves went through a simultaneous gradual reduction of the milk allowance until they were fully weaned at day 60^25^. Each pen also had troughs with concentrates, straw and water for the calves to access ad-libitum.

**Fig. 1:**
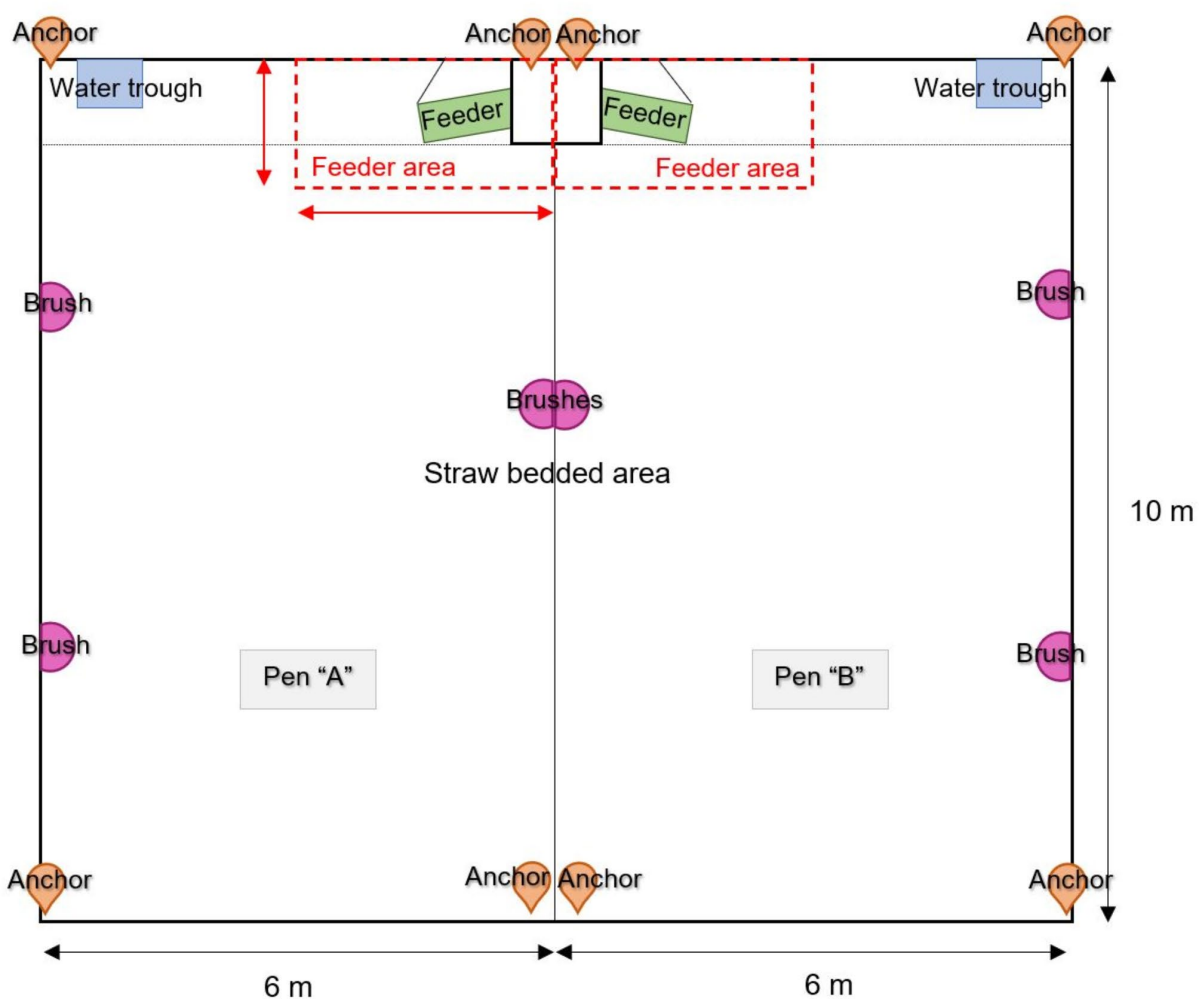
Diagram of the trial pen indicating the position of the location anchors, brushes and feeders.

### Health monitoring

In all analyses (see below), we controlled for calf health status. Calves were inspected twice weekly by a trained researcher for signs of disease using the Wisconsin-Madison calf health scoring system^35^. For the purpose for the trial a calf was considered sick if it has a total score above 4.5 and/or cough score above 2 and/or a temperature above 39.4. The farm staff also visually inspected the calves daily and administered treatments based on farm protocols.

### Experimental brush allocation

Cohort 1–8 were raised as per normal farm management without any form of enrichment and were used as our control group. Starting from cohort 9, the calves were provided with brushes after 10 days of habituation to the trial pen. For cohort 9 onwards, the time in the trial pen was divided into periods of 48 h and each period was randomly assigned to one of the two experimental conditions “brushes absent” or “brushes present”, balancing for the different weaning stages and days with and without health scoring, so that approximately half of the days were assigned to having brushes present. At the start of a period assigned to the “brushes present” condition three stationary brushes were fixed to the walls of each pen as per Fig. 1 and were removed at the end of the period. Each cohort had either 17 or 18 periods of 48 h when the brushes were in the pen (see Table S2 in the supplementary materials for the schedule of brush allocation).

### Sensor data collection

The behavioural data was collected using Ultra-Wideband Sewio Leonardo iMU tags, (Noldus, Wageningen, the Netherlands) housed in counter-weighted collars worn by the calves. The tags recorded the relative coordinates (x, y) of individuals at 1 Hz via triangulation using anchors. The precision and accuracy of the data were validated by keeping the tags in fixed locations and calculating the mean circular error of probability (CEP), calculated as the radius of a circle in which 50% of the datapoints lie, and the mean distance between the ground truth location and the location reported by the sensor (DIST)^36^. The CEP and DIST were 0.15 [0.12–0.28] m and 0.17 [0.13–0.33] m respectively. The calves were recruited at least 7 days prior to being moved to the trial pen to allow for habituation to the collars, followed by 10 days of habituation to the pen before the start of the brush allocation schedule.

### Pre-processing and cleaning of positional data

Any times when people entered the pens were removed from the dataset as well as any points that were reported to be outside of the pen coordinates (representing a total of 7.5% of the data). The data was then smoothed using a moving average over a 10-s window, before movement parameters were computed.

### Behavioural parameters

From the smoothed coordinates it was determined whether each calf was in proximity of the brushes, defined as within 0.5 m from the location of any brush (which is approximately the distance between the tag on the collar and the nose of a calf). From this the following daily measured were computed: total time near the brushes, number of visits near the brushes, and mean visit duration near the brushes. From the same smoothed coordinates, the daily distance travelled (sum of the distance between consecutive points over 24 h for each day) and residence time were computed. Residence time is a measure of how much time an individual spends at the same location, and we used the time spent inside a circle of a 1-m radius centred around its location, without leaving it for more than 1 min, averaged over 24 h^26,37,38^. The total time spent in locomotor play per day was obtained with a two-step classification and quantification algorithm (AdaBoost ensemble learning algorithm and adjusted count technique) applied to feature characteristics of three components of calf movement (speed, turning angle, and turning angle speed) derived from the smoothed coordinates^23^. The adjusted count technique allows us to account for the overestimation generally seen in low-prevalence behaviours. The time that calves spent in the feeder area was computed by counting the number of points that lie within an area of 1.5 m x 3 m surrounding the automatic feeder (Fig. 1). In addition, the feeding information for all the 8 cohorts that did not have brushes and 5 of the cohorts that had brushes, due to a data recording malfunction, was extracted from the automatic feeders and the following daily features were computed: total visits, number of entitled visits, number of unentitled visits, entitled visit duration, number of entitled meals, entitled meal duration, feeding speed^39^ (Table 1).

**Table 1:** Definitions of feeding behaviours computed from the automatic feeder data.

Behavioural parameter	Definition
Number of total visits	Number of times a calf is detected by the feeder as entering and exiting the feeder per day
Number of entitled visits	Number of visits per day when an entitlement of milk replacer was present
Number of unentitled visits	Number of visits per day when an entitlement of milk replacer was not available to the calf
Entitled visit duration	Mean daily duration of visits to the feeder (s)
Number of entitled meals	The daily number of meals per calf, calculated by the visits to the feeder by the same calf that were close in time (< 120 s) when the calf is entitled to milk
Entitled meal duration	The mean duration of entitled meals per calf per day, including any time between consecutive visits if less than 120 s apart (s)
Feeding speed	Mean daily feeding rate (mL/min) calculated by the feeder by dividing the amount of milk (mL) by the visit duration (min) for each visit to the feeder where the calf is entitled to and consumes milk

### Statistical analysis

In the first part of the analysis, we tested the effects of the presence of brushes on the 8 cohorts that had access to them by fitting mixed effects generalised linear models using the lme4^40^ package in R^41^. We fitted one model for each of the behavioural parameters computed, with the parameter as response variables: total time near the brushes, number of visits near the brushes, mean visit duration near the brushes, daily distance travelled, residence time, time spent in locomotor play, number of entitled meals, entitled meal duration, feeding speed, number of entitled visits, number of unentitled visits, entitled visit duration and time spent in the feeder area. In all the models the fixed effects included presence of brushes (absent, present) as our main variable of interest. We controlled for feeding stage (full entitlement, weaning and weaned), health status as defined above (healthy, sick), the experiment day, the age of the calf on the first day in the trial pen and the mean environmental temperature (all continuous effects), and calf ID and cohort number were added as random effects. To test for consistent individual differences in brush use, we calculated the adjusted repeatability (*R*) by extracting the variation due to the random effect and dividing it by the total phenotypic variation to determine the proportion of the total variation that is due to individual identity^42,43^. For models where feeding measures were a response variable, we only computed days in which the calves were entitled to the full milk allowance. In the measures of brush use (total time, number of visits and visit duration near the brushes) we also controlled for time spent along the edges of the pen by adding one of the following as a fixed effect: total time, number of visits and visit duration in the edges of the pen.

In our second set of models, we repeated the above models, but also included 8 cohorts that were never exposed to brushes. We added this condition as an additional fixed effect (no brushes, brushes), while keeping the fixed effect of “brushes present” and “brushes absent” to account for the actual presence of brushes in the pen, and re-run the models with play, daily distance travelled, residence time, time spent in the feeder area and all the feeding measures as the outcome. If the outcome variable was not normally distributed we used a Gamma distribution for continuous variables and Poisson for discrete variables with logarithmic link function.

## Results

### Brush use

**Fig. 2:**
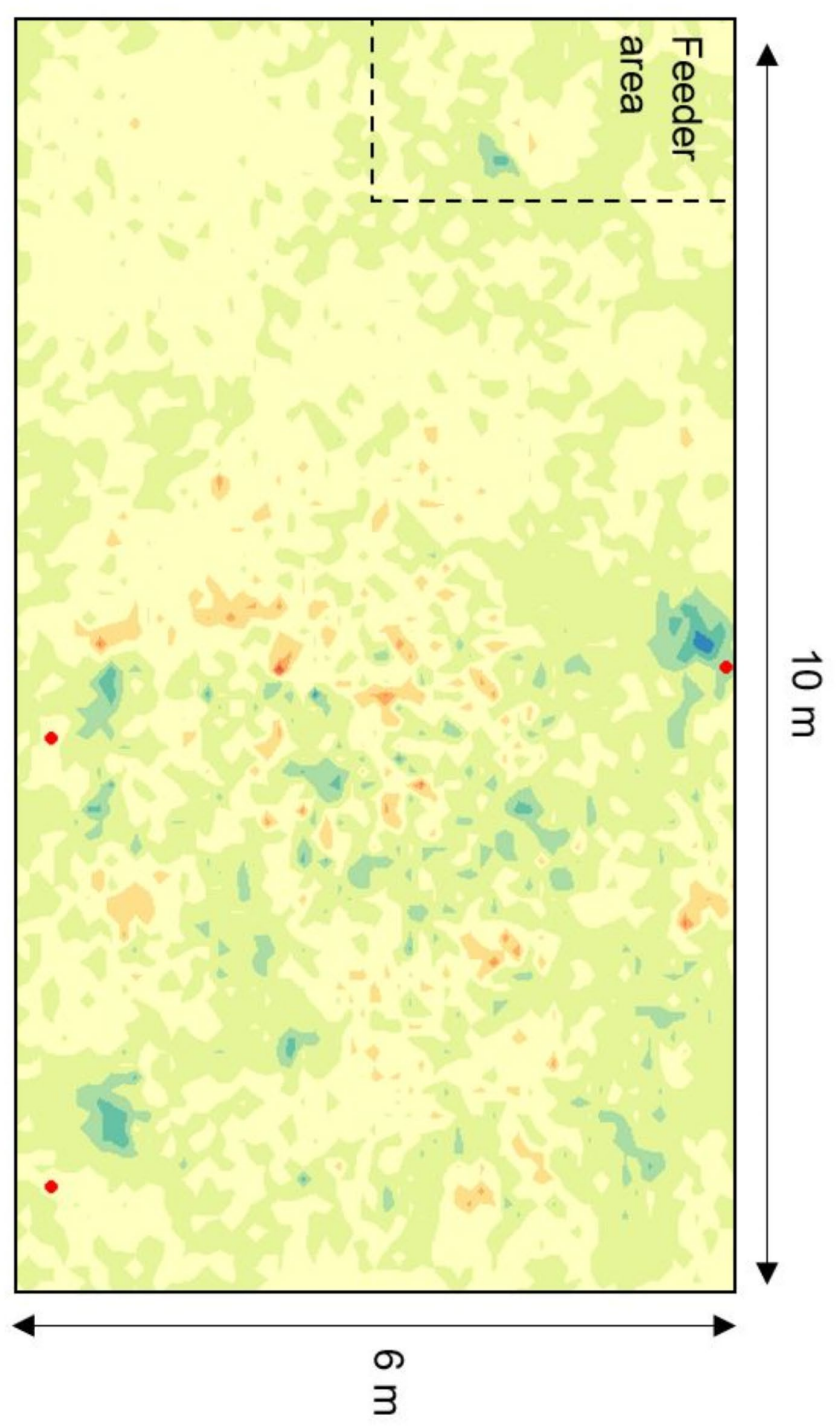
Distribution of the difference between the cumulative space use of all calves in cohort 16 on the days with brushes present and absent. The blue colour represents the areas that have higher use when the brushes are present, and the orange colour represents the areas with lower use. The red dots show the location of each of the three brushes.

Calves spent 19% more time in the areas where the brushes were located when the brushes were present compared to when they were not (β = 0.17, SE = 0.02, *p* < 0.001; Fig. 2). Similarly, calves had 19% more visits (β = 0.18, SE = 0.01, *p* < 0.001) but did not have longer visits to those areas when the brushes were present (β = 0.01, SE = 0.01, *p* = 0.33). The time spent in the brush areas did not change over days (β = -0.04, SE = 0.03, *p* = 0.18) suggesting brush use persisted with no habituation effects during our experimental period, but there was a 6% decrease in the number of visits over time (β = -0.06, SE = 0.02, *p* < 0.001). All measures of brush use had a low repeatability (total time near the brushes: *R* = 0.08 [0.06, 0.09]; number of visits: *R* = 0.10 [0.08, 0.11]; visit duration: *R* = 0.06 [0.05, 0.07], *N* = 101), indicating low inter-individual variation (see supplementary Table S3 for the full model output and estimates).

### Effects of brush presence on calf locomotor play and movement behaviours

Calves increased their distance travelled by 2% (β = 31.29, SE = 9.00, *p* = 0.001) and had a 2% decrease in residence time (β = -0.02, SE = 0.005, *p* < 0.001) on days when the brushes were present compared to days when they were not, but there was no change in the amount of time spent in locomotor play on days with and without brushes (β = 0.01, SE = 0.02, *p* = 0.54) (see supplementary Tables S4-S5 for the full model output and estimates).

### Effects of access to brushes on calf locomotor play and movement behaviours

Including both days with and without brushes, calves from cohorts that had access to brushes had a 53% increase in time spent playing (β = 0.43, SE = 0.11, *p* = 0.004), increased their distance travelled by 28% (β = 427.56, SE = 119.13, *p* = 0.003) and had 14% higher residence time (β = 0.13, SE = 0.02, *p* < 0.001) compared to calves from cohorts that never had access to brushes (see supplementary Tables S6-S7 for the full model output and estimates).

### Effects of brush presence on feeding behaviour

The meal duration decreased by 2% (β = -0.02, SE = 0.01, *p* = 0.04) and the feeding speed decreased by 3% (β = -21.99, SE = 6.98, *p* = 0.002) on days when the brushes were present compared to days when they were not, but there were no significant effects of brush presence on the number of entitled meals (β = -0.002, SE = 0.02, *p* = 0.92). The number of entitled and unentitled visits to the feeder decreased by 19% (β = -0.18, SE = 0.02, *p* < 0.001) and by 7% (β = -0.07, SE = 0.01, *p* < 0.001) respectively on days when the brushes were present compared to days when they were not, while the duration of the entitled visits increased by 9%(β = 16.68, SE = 2.19, *p* < 0.001) (see supplementary Tables S8-S9 for the full model output and estimates).

### Effects of access to brushes on feeding behaviour

The feeding behaviour of the calves was not affected by whether the cohort had access to brushes (including days when the brushes were absent) or were never exposed to it (the effect sizes and model parameters can be found in the supplementary material Tables S10- S11).

### Effects of brush presence on time spent around the feeder

The time that calves spent in the feeder area was 2% lower when the brushes were present compared to the days when the brushes were absent (β = -0.02, SE = 0.01, *p* = 0.02) (see supplementary Table S12 for the full model output and estimates).

## Discussion

We conducted the first study investigating the effects of access to brushes on a wide variety of behaviours including activity, play (using ultra-wideband location sensors) and feeding behaviour (using automatic milk feeders) across hundreds of individuals. Calves used the brushes throughout the trial with limited individual variation between calves. When brushes were present, calves showed increased activity and play, as well as feeding changes including slower drinking and fewer, longer visits. These results demonstrate that brushes can have significant effects on calf behaviours that are not directly linked with the use of the brush and some effects persist even when the brushes are absent. This suggests that brushes act as a form of environmental enrichment.

The total time spent in the brush area and the number of visits in the brush area, were significantly higher when the brushes were present, meaning that the calves were attracted to the brushes as predicted (prediction 1). This engagement by the animals and the lack of habituation to the brushes support the idea that brushes are a source of enrichment for calves^44^. Contrary to our prediction (prediction 3), the low inter-individual variation in the use of the brushes suggests that the use of brushes does not reflect personality differences, which are evident in many other behaviours^22,24,26^. However, this is a positive finding from a welfare perspective since it indicates that all calves used the brushes to a similar degree, and therefore all likely benefitted from the provision of this type of enrichment similarly. Previous work on adult cattle showed differences between dominant and submissive cows in how much time they spent using the rotating brushes, with the dominant individuals using them more overall and especially during peak feeding times^27^. Therefore, it is possible that although young calves do not show individual differences in the overall daily use, individual brush use could vary between calves throughout the day or develop longer-term.

As predicted (prediction 7) the access to brushes was associated with higher activity in calves that experienced it, and even higher activity in the presence of the brushes (prediction 4). This is the first time that the effect of enrichment on the daily distance travelled -a simple metric of activity- was measured in farm animals. The difference was more marked between the cohorts that had access to brushes and those than did not compared to the difference between the days when the brushes were physically in the pen and the days when they were not, indicating that the effect is not merely driven by the calves moving to and from the brush location, but rather a difference in their behaviour possibly driven by a positive affective state. Although the link between higher activity and positive affective states is not yet established, cows are known to be motivated to engage in locomotor activity when they have access to open areas^15^ and being more active is thought to have beneficial effects on health^45^. The fact that in our study higher activity was not limited to the days when the brushes were present, suggests that the positive effects of this type of enrichment persist in time and can cause a potentially long-term change in calf behaviour with possible lasting positive effects for their welfare and health. Note that the first 8 cohorts of calves were all housed without any access to the brushes and the next 8 cohorts had brushes available for half of the trial period. It is possible that this could have introduced an unknown temporal effect, but the management was consistent throughout, and we controlled for daily mean temperature in our analyses.

The cohorts that had access to brushes spent more time playing than ones that did not have access to brushes, but there was no significant difference in the days when the brushes were actually present in the pen. Play behaviour is one of the few suggested indicators of positive welfare and is believed to be associated with positive affective states due to its correlations with health and physical needs being met^29,30^, and motivation to perform this behaviour after being restrained^46^. The fact that the calves that had access to brushes performed more play behaviour, regardless of the presence of brushes on the day, supports the suggestion that the access to the brushes may change the affective state of the calves not only on days when they can use the brush, but also when they are removed, making them more playful throughout the whole study. Future research is needed to confirm whether this can translate to long-lasting effects on their welfare. A previous smaller study, which compared the behaviour of 10 calves in standard hutches to 9 calves who had access to a range of enrichment items, including a brush, showed that the latter calves spent more time performing play behaviour^18^. Our results confirm that exposure to stationary brushes alone can encourage more play behaviour and therefore increase positive welfare, and that the enrichment does not need to be provided continuously for this effect to be noticed.

The residence time was overall higher in cohorts that had access to brushes, which is contrary to our prediction (prediction 4), but decreased slightly when the brushes were in the pen. The higher residence time indicates that the calves were on average spending more time in an area before moving on. This, in conjunction with the higher activity, could indicate a reduction boredom and therefore less pacing and stereotypic behaviours performed^7^. Instead, calves may have performed more directed movement and longer stretches of resting in between due to a more relaxed and content state. However, when the brushes are in the pen the calves may break up their rest periods to go interact with the brushes, which explains the slightly lower residence time. More work is needed to determine what type of behaviours led to this outcome.

Several feeding behaviours were affected by the presence of the brushes in the pen. The calves drank slower when the brushes were present, possibly indicating a more relaxed state induced by the positive experience of using the brushes. Despite this change in behaviour, the calves almost always consumed all their milk allocation, so the change did not affect how many calories they consumed. In a previous study slower drinking speed, which was assessed by altering the teat flow rate, was found to improve lactose digestion and rumen development, meaning that the change caused by the presence of the brushes may benefit nutrient absorption and boost growth rates^47^. The shorter meal duration, combined with fewer visits and longer visit duration, indicates that the meals were less interrupted, as the breaks between visits contributed to the meal duration and therefore fewer interruptions would result in a shorter meal duration. The unentitled visits decreased with the presence of the brushes, which taken alone may indicate a reduction in boredom-induced non-nutritive oral behaviours as was suggested in a previous study^17^. However, in our study the entitled visits were also reduced, and to a greater extent, indicating that calves were using the feeder in longer intervals, and this suggest fewer interruptions as mentioned above. The presence of an alternative activity may lift the competition with other calves that would otherwise linger around the feeder and allow calves to stay in the feeder longer. There were no significant differences between the cohorts that had access to brushes and the ones that did not, meaning that the changes in feeding were limited to the days in which the brushes were present.

As predicted, the calves spent significantly less time in the feeder area when the brushes were present (prediction 5). In our model we controlled for the time spent inside the feeder boundary meaning that this decrease was not due to less time spent using the feeder, but rather due to a change in the time that calves spent around it waiting for other calves to exit it or attempting to actively displace them. As suggested above, in the absence of brushes calves may linger around the feeder as a sign of boredom, as it is the main source of reward in a standard calf enclosure. The reduction in time spent in the feeder area supports the idea that the changes in feeding behaviour associated with the presence of brushes is at least partially caused by the lack of other calves in the area, which reduces the attempted displacements on the calf that is feeding and therefore contributes to the reduced feeding speed, fewer but longer visits and shorter meal duration due to fewer breaks in the meal. These results indicate that the addition of environmental enrichment may decrease the feeding competition that socially housed calves experience^31^, but further studies could measure competition directly to strengthen this conclusion.

Overall, these results confirm the importance of providing sources of environmental enrichment to young calves. The calves in the study used the stationary brushes uniformly and continuously throughout the trial, with limited individual variation or habituation to the item. Our finding that having had brushes in the pen is associated with higher activity is novel in calves and supports the existing evidence from adult cattle. The fact that the difference in activity and in play behaviour were also present regardless of the presence of the brushes on a given day suggests the potential of enrichment to impact behaviour and welfare even after the exposure and future studies should aim to detect the presence of any long-term effects on behaviour or welfare indicators. The effects on feeding behaviour are also novel and especially impactful as the change in feeding speed and reduced interruptions to the meals could impact nutrient absorption and the growth rate of calves, but in this case the change only happened when the brushes were present and may be specific to the feeding method used here. Therefore, this study highlights the complexity of the response to enrichment, which varies depending on the observed behaviour. It also suggests the potential for lasting changes in welfare, as indicated by higher activity and playfulness, as well as suggesting potential physical changes caused by the differences in feeding behaviour. Finally, our use of precision livestock technology proved effective in collecting detailed and extensive data and enabled the detection of the behavioural effects of enrichment.

## Electronic supplementary material

Below is the link to the electronic supplementary material.

Supplementary Material 1

## Data Availability

Data to reproduce the results in this study is available from the corresponding author upon reasonable request.
