# Supplementary material for: Dairy calves provided with environmental enrichment are more active, playful and have fewer feeding interruptions
Source: Sci Rep. 2025 Feb 4;15:4241. doi: 10.1038/s41598-025-88129-7 (PMC11794716; doi:10.1038/s41598-025-88129-7)
Supplement: Supplementary file 1 — Supplementary Material 1 [file 41598_2025_88129_MOESM1_ESM.docx]

Dairy calves provided with environmental enrichment are more active, playful and have fewer feeding interruptions

Francesca Occhiuto^1^*, Jorge A. Vázquez-Diosdado^1^, Matthew Thomas^1^, Emma R. Gayner^1^, Andrew J. King^2^ and Jasmeet Kaler^1^*

^1^School of Veterinary Medicine and Science, University of Nottingham, Sutton Bonington Campus, Leicestershire, LE12 5RD, UK

^2^Department of Biosciences, Faculty of Science and Engineering, Singleton Park Campus, Swansea University, Swansea, SA2 8PP, UK

*[francesca.occhiuto@nottingham.ac.uk](mailto:francesca.occhiuto@nottingham.ac.uk)

*[jasmeet.kaler@nottingham.ac.uk](mailto:jasmeet.kaler@nottingham.ac.uk)

**Table S1** We studied 16 cohorts, tracking the majority or all individuals in each cohort.

| **Cohort** | **Calves tracked** | **Calves not tracked** | **Age of tracked calves on day 0** |
| --- | --- | --- | --- |
| **1** | 16 | 0 | 29.29 |
| **2** | 16 | 0 | 39.69 |
| **3** | 16 | 0 | 36.74 |
| **4** | 16 | 0 | 29.20 |
| **5** | 14 | 2 | 27.09 |
| **6** | 16 | 0 | 32.82 |
| **7** | 15 | 0 | 34.97 |
| **8** | 16 | 0 | 35.51 |
| **9** | 13 | 0 | 43.00 |
| **10** | 13 | 1 | 38.11 |
| **11** | 12 | 0 | 37.79 |
| **12** | 13 | 0 | 38.30 |
| **13** | 10 | 0 | 49.22 |
| **14** | 13 | 1 | 50.17 |
| **15** | 13 | 0 | 35.68 |
| **16** | 14 | 0 | 37.18 |
| **Total** | 226 | 4 | 37.18 |

**Table S2** Brush allocation schedule for cohorts 9-16. The days when the brushes were in the pen are in green and the days when they were absent are in orange.

| **Day** | **Cohort** | | | | | | | |
| --- | --- | --- | --- | --- | --- | --- | --- | --- |
|  | **9** | **10** | **11** | **12** | **13** | **14** | **15** | **16** |
| **1-10** |  |  |  |  |  |  |  |  |
| **11** | ABSENT | ABSENT | ABSENT | ABSENT | ABSENT | ABSENT | PRESENT | ABSENT |
| **12** | ABSENT | ABSENT | ABSENT | ABSENT | ABSENT | ABSENT | PRESENT | ABSENT |
| **13** | PRESENT | ABSENT | ABSENT | PRESENT | ABSENT | ABSENT | PRESENT | PRESENT |
| **14** | PRESENT | ABSENT | ABSENT | PRESENT | ABSENT | ABSENT | PRESENT | PRESENT |
| **15** | PRESENT | PRESENT | PRESENT | PRESENT | ABSENT | PRESENT | ABSENT | ABSENT |
| **16** | PRESENT | PRESENT | PRESENT | PRESENT | ABSENT | PRESENT | ABSENT | ABSENT |
| **17** | ABSENT | PRESENT | PRESENT | PRESENT | PRESENT | PRESENT | PRESENT | ABSENT |
| **18** | ABSENT | PRESENT | PRESENT | PRESENT | PRESENT | PRESENT | PRESENT | ABSENT |
| **19** | PRESENT | ABSENT | ABSENT | ABSENT | PRESENT | PRESENT | PRESENT | ABSENT |
| **20** | PRESENT | ABSENT | ABSENT | ABSENT | PRESENT | PRESENT | PRESENT | ABSENT |
| **21** | PRESENT | ABSENT | ABSENT | ABSENT | PRESENT | PRESENT | PRESENT | ABSENT |
| **22** | PRESENT | ABSENT | ABSENT | ABSENT | PRESENT | PRESENT | PRESENT | ABSENT |
| **23** | ABSENT | ABSENT | PRESENT | ABSENT | ABSENT | ABSENT | ABSENT | ABSENT |
| **24** | ABSENT | ABSENT | PRESENT | ABSENT | ABSENT | ABSENT | ABSENT | ABSENT |
| **25** | PRESENT | ABSENT | PRESENT | ABSENT | PRESENT | ABSENT | ABSENT | ABSENT |
| **26** | PRESENT | ABSENT | PRESENT | ABSENT | PRESENT | ABSENT | ABSENT | ABSENT |
| **27** | ABSENT | PRESENT | ABSENT | ABSENT | ABSENT | PRESENT | ABSENT | PRESENT |
| **28** | ABSENT | PRESENT | ABSENT | ABSENT | ABSENT | PRESENT | ABSENT | PRESENT |
| **29** | PRESENT | ABSENT | PRESENT | ABSENT | PRESENT | PRESENT | PRESENT | PRESENT |
| **30** | PRESENT | ABSENT | PRESENT | ABSENT | PRESENT | PRESENT | PRESENT | PRESENT |
| **31** | ABSENT | PRESENT | ABSENT | ABSENT | PRESENT | ABSENT | PRESENT | PRESENT |
| **32** | ABSENT | PRESENT | ABSENT | ABSENT | PRESENT | ABSENT | PRESENT | PRESENT |
| **33** | ABSENT | PRESENT | PRESENT | PRESENT | PRESENT | ABSENT | PRESENT | PRESENT |
| **34** | ABSENT | PRESENT | PRESENT | PRESENT | PRESENT | ABSENT | PRESENT | PRESENT |
| **35** | ABSENT | PRESENT | PRESENT | PRESENT | ABSENT | ABSENT | ABSENT | PRESENT |
| **36** | ABSENT | PRESENT | PRESENT | PRESENT | ABSENT | ABSENT | ABSENT | PRESENT |
| **37** | PRESENT | PRESENT | ABSENT | PRESENT | ABSENT | PRESENT | PRESENT | PRESENT |
| **38** | PRESENT | PRESENT | ABSENT | PRESENT | ABSENT | PRESENT | PRESENT | PRESENT |
| **39** | PRESENT | ABSENT | ABSENT | ABSENT | PRESENT | PRESENT | ABSENT | PRESENT |
| **40** | PRESENT | ABSENT | ABSENT | ABSENT | PRESENT | PRESENT | ABSENT | PRESENT |
| **41** | ABSENT | ABSENT | PRESENT | PRESENT | ABSENT | ABSENT | PRESENT | PRESENT |
| **42** | ABSENT | ABSENT | PRESENT | PRESENT | ABSENT | ABSENT | PRESENT | PRESENT |
| **43** | ABSENT | PRESENT | ABSENT | ABSENT | PRESENT | PRESENT | ABSENT | PRESENT |
| **44** | ABSENT | PRESENT | ABSENT | ABSENT | PRESENT | PRESENT | ABSENT | PRESENT |
| **45** | ABSENT | PRESENT | PRESENT | ABSENT | ABSENT | ABSENT | PRESENT | ABSENT |
| **46** | ABSENT | PRESENT | PRESENT | ABSENT | ABSENT | ABSENT | PRESENT | ABSENT |
| **47** | ABSENT | ABSENT | PRESENT | PRESENT | ABSENT | ABSENT | PRESENT | ABSENT |
| **48** | ABSENT | ABSENT | PRESENT | PRESENT | ABSENT | ABSENT | PRESENT | ABSENT |
| **49** | PRESENT | PRESENT | PRESENT | PRESENT | PRESENT | PRESENT | ABSENT | PRESENT |
| **50** | PRESENT | PRESENT | PRESENT | PRESENT | PRESENT | PRESENT | ABSENT | PRESENT |
| **51** | ABSENT | PRESENT | PRESENT | ABSENT | PRESENT | ABSENT | ABSENT | ABSENT |
| **52** | ABSENT | PRESENT | PRESENT | ABSENT | PRESENT | ABSENT | ABSENT | ABSENT |
| **53** | PRESENT | PRESENT | ABSENT | ABSENT | PRESENT | ABSENT | PRESENT | ABSENT |
| **54** | PRESENT | PRESENT | ABSENT | ABSENT | PRESENT | ABSENT | PRESENT | ABSENT |
| **55** | PRESENT | ABSENT | ABSENT | PRESENT | ABSENT | PRESENT | ABSENT | ABSENT |
| **56** | PRESENT | ABSENT | ABSENT | PRESENT | ABSENT | PRESENT | ABSENT | ABSENT |
| **57** | PRESENT | ABSENT | ABSENT | PRESENT | PRESENT | ABSENT | ABSENT | PRESENT |
| **58** | PRESENT | ABSENT | ABSENT | PRESENT | PRESENT | ABSENT | ABSENT | PRESENT |
| **59** | PRESENT | PRESENT | ABSENT | ABSENT | PRESENT | ABSENT | PRESENT | PRESENT |
| **60** | PRESENT | PRESENT | ABSENT | ABSENT | PRESENT | ABSENT | PRESENT | PRESENT |
| **61** | ABSENT | PRESENT | PRESENT | ABSENT | ABSENT | PRESENT | PRESENT | PRESENT |
| **62** | ABSENT | PRESENT | PRESENT | ABSENT | ABSENT | PRESENT | PRESENT | PRESENT |
| **63** | ABSENT | ABSENT | ABSENT | PRESENT | PRESENT | PRESENT | PRESENT | ABSENT |
| **64** | ABSENT | ABSENT | ABSENT | PRESENT | PRESENT | PRESENT | PRESENT | ABSENT |
| **65** | PRESENT | ABSENT | ABSENT | PRESENT | PRESENT | PRESENT | PRESENT | ABSENT |
| **66** | PRESENT | ABSENT | ABSENT | PRESENT | PRESENT | PRESENT | PRESENT | ABSENT |
| **67** | PRESENT | PRESENT | ABSENT | ABSENT | ABSENT | PRESENT | ABSENT | ABSENT |
| **68** | PRESENT | PRESENT | ABSENT | ABSENT | ABSENT | PRESENT | ABSENT | ABSENT |
| **69** | ABSENT | ABSENT | ABSENT | ABSENT | ABSENT | ABSENT | ABSENT | PRESENT |
| **70** | ABSENT | ABSENT | ABSENT | ABSENT | ABSENT | ABSENT | ABSENT | PRESENT |
| **71** | PRESENT | ABSENT | PRESENT | ABSENT | ABSENT | PRESENT | ABSENT | ABSENT |
| **72** | PRESENT | ABSENT | PRESENT | ABSENT | ABSENT | PRESENT | ABSENT | ABSENT |
| **73** | PRESENT | ABSENT | PRESENT | PRESENT | PRESENT | PRESENT | ABSENT | PRESENT |
| **74** | PRESENT | ABSENT | PRESENT | PRESENT | PRESENT | PRESENT | ABSENT | PRESENT |
| **75** | PRESENT | PRESENT | ABSENT | PRESENT | PRESENT | ABSENT | ABSENT | PRESENT |
| **76** | PRESENT | PRESENT | ABSENT | PRESENT | PRESENT | ABSENT | ABSENT | PRESENT |
| **77** | ABSENT | PRESENT | PRESENT | PRESENT | ABSENT | PRESENT | ABSENT | PRESENT |
| **78** | ABSENT | PRESENT | PRESENT | PRESENT | ABSENT | PRESENT | ABSENT | PRESENT |
| **79** | ABSENT | ABSENT | PRESENT | ABSENT | ABSENT | ABSENT | ABSENT | ABSENT |
| **80** | ABSENT | ABSENT | PRESENT | ABSENT | ABSENT | ABSENT | ABSENT | ABSENT |
| **81** | ABSENT | PRESENT | PRESENT | PRESENT | ABSENT | ABSENT | PRESENT | ABSENT |
| **82** | ABSENT | PRESENT | PRESENT | PRESENT | ABSENT | ABSENT | PRESENT | ABSENT |

**Table S3** Effect sizes and parameters of the random intercept linear models for each variable of interest: total time, number of visits and visit duration in the brush area, for the cohorts that had access to brushes. In all three models the distribution used was Gamma with logarithmic link function.

|  | | **Total time near brushes** | | | | **Number of visits** | | | | **Visit duration** | | | |
| --- | --- | --- | --- | --- | --- | --- | --- | --- | --- | --- | --- | --- | --- |
| **Fixed effects** | | | | | | | | | | | | | |
|  | Obs. | Est. | St.Error | | p-value | Est. | St.Error | | p-value | Est. | St.Error | | p-value |
| (Intercept) |  | 7.86 | 0.07 | | - | 3.79 | 0.04 | | - | 4.02 | 0.04 | | - |
| Day | 6502 | -0.04 | 0.03 | | 0.18 | -0.06 | 0.02 | | <0.001 | -0.01 | 0.02 | | 0.36 |
| Age day 0 | 6502 | 0.02 | 0.03 | | 0.49 | 0.01 | 0.02 | | 0.57 | -0.005 | 0.02 | | 0.78 |
| Health status - Healthy | 5429 | Ref. | - | | - | Ref. | - | | - | Ref. | - | | - |
| Health status - Sick | 1073 | 0.02 | 0.03 | | 0.39 | -0.003 | 0.02 | | 0.86 | 0.04 | 0.02 | | 0.02 |
| Feeding – full milk | 2408 | Ref. | - | | - | Ref. | - | | - | Ref. | - | | - |
| Feeding – step down | 1838 | -0.01 | 0.04 | | 0.73 | 0.02 | 0.02 | | 0.39 | -0.03 | 0.02 | | 0.26 |
| Feeding – weaned | 2256 | -0.17 | 0.06 | | 0.01 | -0.08 | 0.04 | | 0.05 | -0.11 | 0.04 | | 0.003 |
| Edge areas (time/  bout count) | 6502 | 10.43 | 0.01 | | <0.001 | 0.31 | 0.01 | | <0.001 | 0.20 | 0.01 | | <0.001 |
| Mean temperature | 6502 | 0.02 | 0.01 | | 0.13 | 0.04 | 0.01 | | <0.001 | -0.03 | 0.01 | | 0.003 |
| Brushes - Absent | 3320 | Ref. | - | | - | Ref. | - | | - | Ref. | - | | - |
| *Brushes - Present* | *3182* | *0.17* | *0.02* | | *<0.001* | *0.18* | *0.01* | | *<0.001* | *0.01* | *0.01* | | *0.33* |
| **Random effects** | | | | | | | | | | | | | |
| **Groups** | N | Variance | | St.Dev | | Variance | | St.Dev | | Variance | | St.Dev | |
| Calf (intercept) | 101 | 0.04 | | 0.20 | | 0.01 | | 0.12 | | 0.01 | | 0.09 | |
| Cohort (intercept) | 8 | 0.01 | | 0.11 | | 0.001 | | 0.04 | | 0.002 | | 0.05 | |
| Residual | - | 0.62 | | 0.79 | | 0.24 | | 0.50 | | 0.22 | | 0.47 | |
| Marginal R2 | | 0.19 | | | | 0.28 | | | | 0.15 | | | |
| Conditional R2 | | 0.24 | | | | 0.32 | | | | 0.19 | | | |

**Table S4** Effect sizes and parameters of the random intercept linear models for each variable of interest: distance travelled and residence time, for the cohorts that had access to brushes. The distribution used for the residence time model was Gamma with logarithmic link function.

|  | | **Distance travelled (m)** | | | | **Residence time** | | | |
| --- | --- | --- | --- | --- | --- | --- | --- | --- | --- |
| **Fixed effects** | | | | | | | | | |
|  | Obs. | Est. | St.Error | | p-value | Est. | St.Error | | p-value |
| (Intercept) |  | 1815.41 | 28.39 | | - | 8.43 | 0.02 | | - |
| Day | 6586 | 2.11 | 0.64 | | <0.001 | -0.03 | 0.01 | | <0.001 |
| Age day 0 | 6586 | 34.99 | 22.19 | | 0.12 | -0.03 | 0.02 | | 0.06 |
| Health status - Healthy | 5509 | Ref. | - | | - | Ref. | - | | - |
| Health status - Sick | 1077 | -121.53 | 13.18 | | <0.001 | 0.04 | 0.01 | | <0.001 |
| Feeding – full milk | 2458 | Ref. | - | | - | Ref. | - | | - |
| Feeding – step down | 1835 | -35.37 | 18.75 | | 0.06 | -0.04 | 0.01 | | 0.001 |
| Feeding – weaned | 2293 | -168.72 | 30.94 | | <0.001 | -0.12 | 0.02 | | <0.001 |
| Mean temperature | 6586 | 38.35 | 7.06 | | <0.001 | -0.06 | 0.004 | | <0.001 |
| Brushes - Absent | 3330 | Ref. | - | | - | Ref. | - | | - |
| *Brushes - Present* | *3256* | *31.29* | *9.00* | | *>0.001* | *-0.02* | *0.01* | | *<0.001* |
| **Random effects** | | | | | | | | | |
| **Groups** | N | Variance | | St.Dev | | Variance | | St.Dev | |
| Calf (intercept) | 101 | 48282.00 | | 219.70 | | 0.006 | | 0.07 | |
| Cohort (intercept) | 8 | - | | - | | <0.001 | | <0.001 | |
| Residual | - | 132762.00 | | 364.40 | | 0.05 | | 0.24 | |
| Marginal R2 | | 0.03 | | | | 0.17 | | | |
| Conditional R2 | | 0.30 | | | | 0.25 | | | |

**Table S5** Effect sizes and parameters of the random intercept linear models for total play behaviour, for the cohorts that had access to brushes. In this model the distribution used was Gamma with logarithmic link function.

|  | | **Total play** | | | |
| --- | --- | --- | --- | --- | --- |
| **Fixed effects** | | | | | |
|  | Obs. | Est. | St.Error | | p-value |
| (Intercept) |  | 4.82 | 0.04 | | - |
| Day | 5108 | -0.13 | 0.20 | | <0.001 |
| Age day 0 | 5108 | -0.08 | 0.03 | | 0.002 |
| Health status - Healthy | 4252 | Ref. | - | | - |
| Health status - Sick | 856 | -0.20 | 0.02 | | <0.001 |
| Feeding – full milk | 1922 | Ref. | - | | - |
| Feeding – step down | 1445 | -0.10 | 0.04 | | 0.003 |
| Feeding – weaned | 1741 | -0.16 | 0.06 | | 0.01 |
| Mean temperature | 5108 | -0.01 | 0.01 | | 0.36 |
| Brushes - Absent | 2640 | Ref. | - | | - |
| *Brushes - Present* | 2468 | 0.01 | 0.02 | | *0.54* |
| **Random effects** | | | | | |
| **Groups** | N | Variance | | St.Dev | |
| Calf (intercept) | 101 | 0.02 | | 0.17 | |
| Cohort (intercept) | 8 | <0.001 | | <0.001 | |
| Residual | - | 0.37 | | 3.17 | |
| Marginal R2 | | 0.09 | | | |
| Conditional R2 | | 0.14 | | | |

**Table S6** Effect sizes and parameters of the random intercept linear models for each variable of interest: distance travelled and residence time, for all the cohorts, including the ones that had access to brushes and the ones that did not. The distribution used for the residence time model was Gamma with logarithmic link function.

|  | | **Distance travelled (m)** | | | | **Residence time** | | | | |  |
| --- | --- | --- | --- | --- | --- | --- | --- | --- | --- | --- | --- |
| **Fixed effects** | | | | | | | | | | |  |
|  | Obs. | Est. | St.Error | | p-value | | Est. | St.Error | | p-value | |
| (Intercept) |  | 1497.10 | 84.29 | | - | | 8.27 | 0.02 | | - | |
| Day | 13918 | -1.32 | 0.41 | | 0.002 | | -0.04 | 0.005 | | <0.001 | |
| Age day 0 | 13918 | 22.13 | 16.44 | | 0.18 | | -0.03 | 0.01 | | 0.003 | |
| Health status - Healthy | 11895 | Ref. | - | | - | | Ref. | - | | - | |
| Health status - Sick | 2023 | -143.75 | 8.86 | | <0.001 | | 0.05 | 0.01 | | <0.001 | |
| Feeding – full milk | 5531 | Ref. | - | | - | | Ref. | - | | - | |
| Feeding – step down | 4207 | -8.54 | 11.86 | | 0.47 | | 0.01 | 0.01 | | 0.10 | |
| Feeding – weaned | 4180 | -53.87 | 19.43 | | 0.01 | | -0.05 | 0.01 | | <0.001 | |
| Mean temperature | 13918 | 27.82 | 4.53 | | <0.001 | | -0.06 | 0.003 | | <0.001 | |
| Access to brushes - No | 7332 | Ref. | - | | - | | Ref. | - | | - | |
| *Access to brushes - Yes* | *6586* | *427.56* | *119.13* | | *0.003* | | *0.13* | *0.02* | | *<0.001* | |
| Brushes - Absent | 10662 | Ref. | - | | - | | Ref. | - | | - | |
| Brushes - Present | 3256 | 32.44 | 8.27 | | <0.001 | | -0.02 | 0.01 | | <0.001 | |
| **Random effects** | | | | | | | | | | |  |
| **Groups** | N | Variance | | St.Dev | | Variance | | | St.Dev | |  |
| Calf (intercept) | 226 | 38518.00 | | 196.30 | | 0.01 | | | 0.11 | |  |
| Cohort (intercept) | 16 | 53096.00 | | 230.40 | | - | | | - | |  |
| Residual | - | 112054.00 | | 334.70 | | 0.05 | | | 0.21 | |  |
| Marginal R2 | | 0.22 | | | | 0.13 | | | | |  |
| Conditional R2 | | 0.57 | | | | 0.33 | | | | |  |

**Table S7** Effect sizes and parameters of the random intercept linear models for total play behaviour for all the cohorts, including the ones that had access to brushes and the ones that did not. The distribution used for this model was Gamma with logarithmic link function.

|  | | **Total play** | | | |
| --- | --- | --- | --- | --- | --- |
| **Fixed effects** | | | | | |
|  | Obs. | Est. | St.Error | | p-value |
| (Intercept) |  | 4.44 | 0.08 | | - |
| Day | 11293 | -0.20 | 0.02 | | <0.001 |
| Age day 0 | 11293 | -0.06 | 0.02 | | 0.01 |
| Health status - Healthy | 9644 | Ref. | - | | - |
| Health status - Sick | 1649 | -0.27 | 0.02 | | <0.001 |
| Feeding – full milk | 4513 | Ref. | - | | - |
| Feeding – step down | 3544 | -0.13 | 0.02 | | <0.001 |
| Feeding – weaned | 3236 | -0.05 | 0.04 | | 0.26 |
| Mean temperature | 11293 | -0.06 | 0.01 | | <0.001 |
| Access to brushes - No | 6185 | Ref. | - | | - |
| *Access to brushes - Yes* | 5108 | *0.43* | *0.11* | | *<0.001* |
| Brushes - Absent | 8825 | Ref. | - | | - |
| Brushes - Present | 2468 | -0.01 | 0.02 | | 0.65 |
| **Random effects** | | | | | |
| **Groups** | N | Variance | | St.Dev | |
| Calf (intercept) | 226 | 0.03 | | 0.18 | |
| Cohort (intercept) | 16 | 0.02 | | 0.13 | |
| Residual | - | 0.40 | | 0.63 | |
| Marginal R2 | | 0.14 | | | |
| Conditional R2 | | 0.22 | | | |

**Table S8** Effect sizes and parameters of the random intercept linear models for each variable of interest: number of entitled meals, entitled meal duration and feeding speed, for the cohorts that had access to brushes. The distribution used for the entitled meals and entitled meal duration models was Gamma with logarithmic link function.

|  | | **Number of entitled meals** | | | | **Entitled meal duration** | | | | **Feed speed (ml/min)** | | | |
| --- | --- | --- | --- | --- | --- | --- | --- | --- | --- | --- | --- | --- | --- |
| **Fixed effects** | | | | | | | | | | | | | |
|  | Obs. | Est. | St.Error | | p-value | Est. | St.Error | | p-value | Est. | St.Error | | p-value |
| (Intercept) |  | 1.69 | 0.05 | | - | 5.43 | 0.07 | | - | 795.63 | 59.37 | | - |
| Day | 1240 | 0.02 | 0.01 | | 0.13 | -0.02 | 0.01 | | <0.001 | 0.79 | 0.44 | | 0.07 |
| Age day 0 | 1240 | -0.02 | 0.01 | | 0.12 | -0.02 | 0.03 | | 0.43 | 30.40 | 12.06 | | 0.01 |
| Health status - Healthy | 936 | Ref. | - | | - | Ref. | - | | - | Ref. | - | | - |
| Health status - Sick | 304 | 0.03 | 0.03 | | 0.33 | -0.01 | 0.01 | | 0.32 | -29.75 | 9.06 | | 0.001 |
| Mean temperature | 1240 | 0.05 | 0.03 | | 0.04 | -0.04 | 0.01 | | <0.001 | -44.18 | 6.55 | | <0.001 |
| Brushes - Absent | 697 | Ref. | - | | - | Ref. | - | | - | Ref. | - | | - |
| *Brushes - Present* | *543* | *-0.002* | *0.02* | | *0.92* | *-0.02* | *0.01* | | *0.04* | *-21.99* | *6.98* | | *0.002* |
| **Random effects** | | | | | | | | | | | | | |
| **Groups** | N | Variance | | St.Dev | | Variance | | St.Dev | | Variance | | St.Dev | |
| Calf (intercept) | 65 | <0.001 | | <0.001 | | 0.01 | | 0.09 | | 8349.00 | | 91.37 | |
| Cohort (intercept) | 5 | 0.005 | | 0.07 | | 0.002 | | 0.04 | | 16359.00 | | 127.90 | |
| Residual | - | 0.06 | | 0.24 | | 0.04 | | 0.19 | | 14203.00 | | 119.17 | |
| Marginal R2 | | 0.02 | | | | 0.04 | | | | 0.08 | | | |
| Conditional R2 | | 0.04 | | | | 0.25 | | | | 0.66 | | | |

**Table S9** Effect sizes and parameters of the random intercept linear models for each variable of interest: number of entitled visits, number of unentitled visits and entitled visit duration, for the cohorts that had access to brushes. The distribution used for the entitled and unentitled visits models was Gamma with logarithmic link function.

|  | | **Number of entitled visits** | | | | **Number of unentitled visits** | | | | **Entitled visit duration** | | | |
| --- | --- | --- | --- | --- | --- | --- | --- | --- | --- | --- | --- | --- | --- |
| **Fixed effects** | | | | | | | | | | | | | |
|  | Obs. | Est. | St.Error | | p-value | Est. | St.Error | | p-value | Est. | St.Error | | p-value |
| (Intercept) |  | 2.09 | 0.16 | | - | 3.43 | 0.18 | | - | 172.82 | 9.27 | | - |
| Day | 1244 | 0.12 | 0.01 | | <0.001 | -0.03 | 0.005 | | <0.001 | -1.12 | 0.14 | | <0.001 |
| Age day 0 | 1244 | -0.02 | 0.02 | | 0.45 | 0.08 | 0.07 | | 0.21 | -5.20 | 3.01 | | 0.09 |
| Health status - Healthy | 938 | Ref. | - | | - | Ref. | - | | - | Ref. | - | | - |
| Health status - Sick | 306 | -0.04 | 0.03 | | 0.09 | -0.10 | 0.01 | | <0.001 | 6.77 | 2.83 | | 0.02 |
| Mean temperature | 1244 | 0.06 | 0.02 | | 0.001 | -0.06 | 0.01 | | <0.001 | 0.53 | 2.03 | | 0.79 |
| Brushes - Absent | 697 | Ref. | - | | - | Ref. | - | | - |  |  | |  |
| *Brushes - Present* | *547* | *-0.18* | *0.02* | | *<0.001* | *-0.07* | *0.01* | | *<0.001* | *16.74* | *2.20* | | *<0.001* |
| **Random effects** | | | | | | | | | | | | | |
| **Groups** | N | Variance | | St.Dev | | Variance | | St.Dev | | Variance | | St.Dev | |
| Calf (intercept) | 65 | 0.03 | | 0.17 | | 0.28 | | 0.52 | | 489.60 | | 22.13 | |
| Cohort (intercept) | 5 | 0.12 | | 0.35 | | 0.14 | | 0.38 | | 330.20 | | 18.17 | |
| Residual | - | - | | - | | - | | - | | 1406.50 | | 37.50 | |
| Marginal R2 | | 0.07 | | | | 0.03 | | | | 0.07 | | | |
| Conditional R2 | | 0.56 | | | | 0.94 | | | | 0.41 | | | |

**Table S10** Effect sizes and parameters of the random intercept linear models for each variable of interest: number of entitled meals, entitled meal duration and feeding speed, for all the cohorts, including the ones that had access to brushes and the ones that did not. The distribution used for the entitled meals and entitled meal duration models was Gamma with logarithmic link function.

|  | | **Number of entitled meals** | | | | **Entitled meal duration** | | | | **Feed speed (ml/min)** | | | |
| --- | --- | --- | --- | --- | --- | --- | --- | --- | --- | --- | --- | --- | --- |
| **Fixed effects** | | | | | | | | | | | | | |
|  | Obs. | Est. | St.Error | | p-value | Est. | St.Error | | p-value | Est. | St.Error | | p-value |
| (Intercept) |  | 1.77 | 0.02 | | - | 5.40 | 0.05 | | - | 768.91 | 29.08 | | - |
| Day | 3579 | -0.01 | 0.007 | | 0.15 | -0.02 | 0.003 | | <0.001 | 0.88 | 0.26 | | <0.001 |
| Age day 0 | 3579 | -0.01 | 0.01 | | 0.72 | -0.005 | 0.02 | | 0.77 | 23.53 | 7.05 | | 0.001 |
| Health status - Healthy | 3061 | Ref. | - | | - | Ref. | - | | - | Ref. | - | | - |
| Health status - Sick | 518 | 0.03 | 0.03 | | 0.13 | -0.01 | 0.01 | | 0.15 | -10.16 | 6.60 | | 0.13 |
| Mean temperature | 3579 | -0.01 | 0.03 | | 0.65 | -0.001 | 0.01 | | 0.82 | -14.49 | 4.13 | | <0.001 |
| Access to brushes - No | 2304 | Ref. | - | | - | Ref. | - | | - | Ref. | - | | - |
| *Access to brushes - Yes* | *1275* | *-0.02* | *0.04* | | *0.56* | *0.05* | *0.06* | | *0.40* | *6.67* | *46.29* | | *0.89* |
| Brushes - Absent | 3000 | Ref. | - | | - | Ref. | - | | - | Ref. | - | | - |
| Brushes - Present | 579 | 0.02 | 0.02 | | 0.48 | -0.02 | 0.01 | | 0.03 | -21.41 | 6.71 | | 0.001 |
| **Random effects** | | | | | | | | | | | | | |
| **Groups** | N | Variance | | St.Dev | | Variance | | St.Dev | | Variance | | St.Dev | |
| Calf (intercept) | 188 | 0.004 | | 0.06 | | 0.01 | | 0.10 | | 6454.00 | | 80.34 | |
| Cohort (intercept) | 13 | 0.003 | | 0.06 | | 0.01 | | 0.03 | | 5974.00 | | 77.29 | |
| Residual | - | 0.07 | | 0.26 | | 0.04 | | 0.19 | | 13791.00 | | 117.43 | |
| Marginal R2 | | 0.004 | | | | 0.01 | | | | 0.03 | | | |
| Conditional R2 | | 0.09 | | | | 0.24 | | | | 0.49 | | | |

**Table S11** Effect sizes and parameters of the random intercept linear models for each variable of interest: number of entitled visits, number of unentitled visits and entitled visit duration, for the cohorts that had access to brushes. The distribution used for the entitled and unentitled visits models was Gamma with logarithmic link function.

|  | | **Number of entitled visits** | | | | **Number of unentitled visits** | | | | **Entitled visit duration** | | | |
| --- | --- | --- | --- | --- | --- | --- | --- | --- | --- | --- | --- | --- | --- |
| **Fixed effects** | | | | | | | | | | | | | |
|  | Obs. | Est. | St.Error | | p-value | Est. | St.Error | | p-value | Est. | St.Error | | p-value |
| (Intercept) |  | 2.01 | 0.09 | | - | 3.40 | 0.12 | | - | 163.19 | 7.45 | | - |
| Day | 3617 | 0.05 | 0.01 | | 0.001 | -0.05 | 0.00 | | <0.001 | -0.48 | 0.08 | | <0.001 |
| Age day 0 | 3617 | -0.01 | 0.01 | | 0.60 | 0.06 | 0.04 | | 0.13 | -2.45 | 1.73 | | 0.16 |
| Health status - Healthy | 3097 | Ref. | - | | - | Ref. | - | | - | Ref. | - | | - |
| Health status - Sick | 520 | -0.01 | 0.02 | | 0.63 | -0.02 | 0.01 | | 0.01 | 4.21 | 2.11 | | 0.05 |
| Mean temperature | 3617 | 0.004 | 0.01 | | 0.77 | -0.05 | 0.01 | | <0.001 | 1.71 | 1.32 | | 0.19 |
| Access to brushes - No | 2331 | Ref. | - | | - | Ref. | - | | - | Ref. | - | | - |
| *Access to brushes - Yes* | *1286* | *0.05* | *0.14* | | *0.73* | *-0.03* | *0.20* | | *0.90* | *-2.58* | *11.72* | | *0.83* |
| Brushes - Absent | 3035 | Ref. | - | | - | Ref. | - | | - | Ref. | - | | - |
| Brushes - Present | 582 | -0.14 | 0.02 | | <0.001 | -0.07 | 0.01 | | <0.001 | 15.11 | 2.17 | | <0.001 |
| **Random effects** | | | | | | | | | | | | | |
| **Groups** | N | Variance | | St.Dev | | Variance | | St.Dev | | Variance | | St.Dev | |
| Calf (intercept) | 188 | 0.01 | | 0.11 | | 0.25 | | 0.50 | | 353.00 | | 18.79 | |
| Cohort (intercept) | 13 | 0.06 | | 0.25 | | 0.10 | | 0.31 | | 383.90 | | 19.59 | |
| Residual | - | - | | - | | 0.39 | | 0.62 | | 1444.1 | | 38.00 | |
| Marginal R2 | | 0.01 | | | | 0.02 | | | | 0.02 | | | |
| Conditional R2 | | 0.36 | | | | 0.93 | | | | 0.35 | | | |

**Table S12** Effect sizes and parameters of the random intercept linear models time in the feeder area for the cohorts that had access to brushes.

|  | | **Time in the feeder area** | | | |
| --- | --- | --- | --- | --- | --- |
| **Fixed effects** | | | | | |
|  | Obs. | Est. | St.Error | | p-value |
| (Intercept) |  | 8.12 | 0.03 | | - |
| Day | 2185 | 0.01 | 0.00 | | 0.15 |
| Age day 0 | 2185 | -0.02 | 0.02 | | 0.44 |
| Health status - Healthy | 1726 | Ref. | - | | - |
| Health status - Sick | 459 | 8.12 | 0.03 | | 0.22 |
| Mean temperature | 2185 | 0.01 | 0.00 | | 0.53 |
| Brushes - Absent | 1138 | Ref. | - | | - |
| *Brushes - Present* | *1047* | -0.02 | 0.01 | | *0.02* |
| Feeder time | 2185 | 0.33 | 0.01 | | <0.001 |
| **Random effects** | | | | | |
| **Groups** | N | Variance | | St.Dev | |
| Calf (intercept) | 100 | 0.01 | | 0.09 | |
| Cohort (intercept) | 8 | <0.001 | | 0.02 | |
| Residual | - | 0.05 | | 0.22 | |
| Marginal R2 | | 0.64 | | | |
| Conditional R2 | | 0.70 | | | |
